# Evaluating Professional Burnout and Psychological Distress Among Intensive Care Unit Healthcare Workers During the COVID-19 Pandemic: A Single-Center Cross-Sectional Study

**DOI:** 10.7759/cureus.81416

**Published:** 2025-03-29

**Authors:** Jonathan Y Boey, Bridget Ng, Yi Lin Lee

**Affiliations:** 1 Medicine, National University Hospital, Singapore, SGP; 2 Anaesthesiology, Singapore General Hospital, Singapore, SGP

**Keywords:** anxiety, covid-19, critical care, depression, intensive care unit, pandemic, professional burnout

## Abstract

Aim

This study aimed to evaluate burnout, stress, anxiety, and depression among intensive care unit (ICU) healthcare workers (HCWs) in Singapore during the COVID-19 pandemic. It sought to identify risk factors associated with burnout to inform targeted interventions and improve HCWs' well-being and patient care quality.

Subject and methods

A cross-sectional survey was conducted in December 2021 in the ICU of Sengkang General Hospital, Singapore. Burnout was measured using the Maslach Burnout Inventory-Human Services Survey (MBI-HSS), stress was assessed with the Cohen Perceived Stress Scale (CPSS), and anxiety and depression were evaluated using the Patient Health Questionnaire-4 (PHQ-4). Physical questionnaires were distributed anonymously to HCWs. Multiple logistic regression analysis was performed to examine associations between burnout and demographic/work-related factors.

Results

A total of 76 HCWs responded to the survey out of 100 who were eligible. The study found an overall burnout rate of 43/76 (56.6%) among HCWs, with respiratory therapists being the most affected. Emotional exhaustion was reported by 53/76 (69.7%) participants, depersonalization by 35/76 (46.1%), and reduced personal accomplishment by 64/76 (84.2%). Multiple logistic regression demonstrated higher burnout rates associated with respiratory therapists and lower burnout rates associated with Filipino ethnicity. Stress affected 66/76 (86.8%) of HCWs, with physicians reporting the highest levels. Stress showed moderate correlations with emotional exhaustion (0.38) and depersonalization (0.33). Anxiety and depression, which were present in 63/76 (82.9%) participants, were strongly correlated with all burnout dimensions.

Conclusion

The findings highlight the urgent need for mental health resources and stress management interventions to address burnout among ICU HCWs, particularly in the context of the COVID-19 pandemic. Implementing targeted strategies to mitigate burnout can enhance HCWs' well-being and maintain high standards of patient care. Further longitudinal research is recommended to explore the long-term impacts and effectiveness of interventions.

## Introduction

Burnout is recognized as a syndrome resulting from unmanaged chronic workplace stress, classified in the International Classification of Diseases, 11th Revision (ICD-11) as an occupational phenomenon [1]. Defined by Maslach and Jackson, burnout encompasses three dimensions: emotional exhaustion (EE), depersonalization (DP), and feelings of diminished personal accomplishment (PA) [2].

EE is characterized by feelings of being emotionally overextended and depleted of emotional resources. DP refers to an unfeeling and impersonal response toward recipients of one's care or service. PA is the sense of competence and successful achievement in one's work. Reduced PA involves feelings of inefficacy and a lack of achievement and productivity at work, contributing to a sense of failure.

In healthcare settings, burnout has long been known to affect personal well-being and professional standards, correlating with lower patient satisfaction, diminished care quality, and increased medical errors and potential malpractice suits [3,4]. On an individual level, burnout is linked to a range of mental and physical health issues, including anxiety, depression, suicidality, substance abuse, and cardiovascular disease [5,6].

Stress, anxiety, and depression among healthcare workers (HCWs) during the COVID-19 pandemic are also closely interrelated and significantly contribute to burnout, in the domains of EE, DP, and PA [7-9].

Despite its serious implications, burnout remains a widespread and under-recognized issue, affecting an estimated 30-50% of clinicians [10], with rates of 15-71% reported among intensive care unit (ICU) staff [11].

The COVID-19 pandemic, which was declared on March 11, 2020, introduced unprecedented challenges to healthcare systems worldwide. ICUs were at the forefront of the crisis, playing a pivotal role in managing critically ill COVID-19 patients. However, the sudden and overwhelming influx of patients, coupled with the challenges of treating a novel and highly contagious virus, placed immense strain on ICU resources and staff. HCWs faced prolonged working hours, shortages of essential medical equipment, and the moral injury sustained from making difficult triage decisions, often under conditions of extreme uncertainty [12]. These challenges were further compounded by the fear of personal infection, loss of colleagues to the virus, and the social isolation resulting from stringent infection control measures.

The psychological impact on HCWs during this period was profound, with many studies showing high rates of psychological symptoms such as burnout, stress, anxiety, depression, post-traumatic stress disorder, and sleep loss among HCWs during the COVID-19 pandemic [13-15].

Despite the global nature of the COVID-19 pandemic, local factors may still lead to variations in patterns and prevalence of burnout and psychological distress across different countries and different healthcare settings. Existing studies done in Singapore have examined these issues in HCWs in general [16,17], but specific insight into a critical care setting may be limited. The findings of this study targeted at ICU HCWs in Singapore may provide valuable insights to enhance the resilience of healthcare systems and safeguard the mental health of those on the frontlines of critical care.

The primary objective was to evaluate the rate of professional burnout among ICU HCWs in Singapore. The secondary objective was to assess contributing factors such as stress, anxiety, depression, and various demographic factors.

## Materials and methods

Study design

This study was a single-center, cross-sectional survey that recruited staff working in the ICU of Sengkang General Hospital (SKH) via convenience sampling. This paper utilized the Strengthening the Reporting of Observational Studies in Epidemiology (STROBE) cross-sectional reporting guidelines [18]. A standardized questionnaire was administered to doctors, nurses, and allied healthcare professionals (AHPs). The survey included demographic questions and three validated psychometric instruments: the Maslach Burnout Inventory-Human Services Survey (MBI-HSS) for assessing burnout, the Cohen Perceived Stress Scale (CPSS) for measuring stress, and the Patient Health Questionnaire-4 (PHQ-4) for evaluating anxiety and depression.

Setting

The survey was conducted in a major regional hospital in Singapore with a 1,000-bed capacity and a 20-bed mixed medical-surgical intensive care unit. Data collection occurred from 1st December 2021 to 31st December 2021, approximately 24 months into the global pandemic and 18 months after lockdown measures began.

Participants

Inclusion criteria for this study encompassed all healthcare professionals working in the ICU, including doctors, nurses, AHPs, and administrative staff. Participants were required to be actively involved in ICU operations and able to complete the questionnaire in English.

Staff not working in the ICU, including visiting staff with no sustained involvement in ICU patient care, were excluded.

Participation was entirely voluntary, and anonymity was assured. The questionnaire was administered in English.

Validated questionnaires

The MBI-HSS consists of 22 items assessed on a seven-point Likert scale across three domains: emotional exhaustion (nine items), depersonalization (five items), and personal accomplishment (eight items). It is the most commonly used and validated tool to detect burnout in clinicians, so it was considered the gold standard [19].

The CPSS includes 10 items rated on a five-point Likert scale, with a score of ≥20 indicating a high perceived stress level [20].

The PHQ-4, a four-item questionnaire rated on a four-point Likert scale, assesses anxiety and depression, with scores of ≥ three indicating possible anxiety or depression [21].

Data collection process

Participants were informed of the study’s purpose through a department-wide briefing, and physical questionnaire forms were distributed. Completed surveys were submitted anonymously into a secure box. Ethics approval was waived (Institutional Review Board Reference Number: 2020/2615), and no risks were posed to participants, who participated voluntarily without incentives. Data collection was stopped at one month due to resource limitations.

Statistical methods

Multiple logistic regression was conducted to examine the associations between professional burnout, assessed using the MBI-HSS, and various demographic and occupational factors. Independent variables included age, gender, race, religion, housing type, marital status, educational level, job role, years of experience, shift patterns, and working hours per week. Odds ratios (ORs) with 95% confidence intervals (CIs) were reported to determine the strength and significance of associations.

Perceived stress, anxiety, and depression were assessed as secondary outcome measures using the Perceived Stress Scale (PSS), Generalized Anxiety Disorder-7 (GAD-7), and Patient Health Questionnaire-9 (PHQ-9), respectively. Univariate analyses were initially performed using chi-square tests for categorical variables and independent t-tests or Mann-Whitney U tests for continuous variables, depending on the normality of distribution (assessed via the Shapiro-Wilk test).

Statistically significant variables (p < 0.05) from univariate analyses were included in multivariable logistic regression models, adjusting for potential confounders. Model fit was assessed using the Hosmer-Lemeshow goodness-of-fit test, and variance inflation factors (VIFs) were examined to check for multicollinearity among independent variables. Sensitivity analyses were conducted to ensure the robustness of findings.

All statistical analyses were performed using R version 2024.09.0+375 (R Foundation for Statistical Computing, Vienna, Austria) with the tidyverse and stats packages. A two-tailed p-value of <0.05 was considered statistically significant.

## Results

Participants

A response rate of 76/100 (76%) was achieved. Of the participants, 49/76 (64.5%) were nurses, 15/76 (19.7%) were doctors, 10/76 (13.2%) were respiratory therapists, and 2/76 (13.2%) were patient service staff. Participant demographic data are presented in Table 1.

**Table 1:** Demographics of study population, including age, gender, ethnicity, and type of housing. Values are presented as raw numbers with the percentage of total respondents in parentheses.

Demographic	Total (n = 76)	Nurse (n = 49)	Doctor (n = 15)	Respiratory therapists (n = 10)	Patient service staff (n = 2)
Age					
21-30	39 (51.3%)	26 (53.1%)	8 (53.3%)	4 (40%)	1 (50%)
31-40	29 (38.2%)	20 (40.8%)	6 (40.0%)	3 (30%)	0 (0%)
41-50	7 (9.2%)	3 (6.1%)	1 (6.7%)	3 (30%)	0 (0%)
51-60	0 (0%)	0 (0%)	0 (0%)	0 (0%)	0 (0%)
61-70	1 (1.3%)	0 (0%)	0 (0%)	0 (0%)	1 (50%)
Gender					
Female	58 (76.3%)	37 (75.5%)	10 (66.7%)	9 (90%)	2 (100%)
Male	18 (23.7%)	12 (24.5%)	5 (33.3%)	1 (10%)	0 (0%)
Ethnicity					
Chinese	43 (56.6%)	24 (49.0%)	14 (93.3%)	5 (50%)	0 (0%)
Malay	10 (13.2%)	10 (20.4%)	0 (0%)	0 (0%)	0 (0%)
Indian	5 (6.6%)	2 (4.1%)	1 (6.7%)	0 (0%)	2 (100%)
Filipino	18 (23.7%)	13 (26.5%)	0 (0%)	5 (50%)	0 (0%)
Housing					
Public housing	62 (81.6%)	43 (87.8%)	8 (53.3%)	9 (90%)	2 (100%)
Condominium	7 (9.2%)	3 (6.1%)	3 (20.0%)	1 (10%)	0 (0%)
Landed housing	7 (9.2%)	3 (6.1%)	4 (26.7%)	0 (0%)	0 (0%)

Outcome data

Burnout Assessment (MBI-HSS)

The study revealed an overall burnout rate of 43/76 (56.6%) among HCWs. AHPs exhibited the highest burnout risk at 8/10 (80.0%), followed by nurses at 27/49 (55.1%) and physicians at 8/15 (53.3%). However, these differences were not statistically significant (p = 0.739).

For DP, the prevalence was 35/76 (46.1%), with a mean score of 11.5 (95% CI: 9.70-13.3, p = 0.742), showing no significant variation across professions. PA burnout was notably high at 64/76 (84.2%), with a mean score of 29.1 (95% CI: 26.8-31.4, p = 0.424), also showing no significant differences among groups. Examining EE, 53/76 (69.7%) experienced burnout, with a mean score of 32.9 (95% CI: 30.4-35.3, p = 0.330). Rates of EE burnout were similar across groups. Table 2 summarizes the results of the MBI-HSS by occupation.

**Table 2:** Results of MBI-HSS survey by occupation showing highest rates of burnout in respiratory therapists. MBI-HSS: Maslach Burnout Inventory-Human Services Survey, used for assessing burnout. Values are presented as raw numbers, with the percentage of total respondents in parentheses.

MBI-HSS items	Total (n = 76)	Nurses (n = 49)	Doctors (n = 15)	Respiratory therapist (n = 10)	Patient service staff (n = 2)
Emotional exhaustion					
Yes	53 (69.7%)	37 (75.5%)	9 (60.0%)	7 (70%)	0 (0%)
No	23 (30.3%)	12 (24.5%)	6 (40.0%)	3 (30%)	2 (100%)
Lack of personal accomplishment					
Yes	64 (84.2%)	41 (83.7%)	14 (93.3%)	9 (75%)	0 (0%)
No	12 (15.8%)	8 (16.3%)	1 (6.7%)	3 (25%)	2 (100%)
Depersonalization					
Yes	35 (46.1%)	21 (42.9%)	8 (53.3%)	6 (60%)	0 (0%)
No	41 (53.9%)	28 (57.1%)	7 (42.9%)	4 (40%)	2 (100%)
Overall burnout					
Yes	43 (56.6%)	27 (55.1%)	8 (53.3%)	8 (80%)	0 (0%)
No	33 (43.4%)	22 (44.9%)	7 (46.7%)	2 (20%)	2 (100%)

Multiple logistic regression was performed to look at the factors associated with burnout in Table 3. Significantly, respiratory therapists showed increased rates of burnout (p = 0.024), and Filipino ethnicity was associated with decreased rates of burnout (p = 0.0404). There were no significant associations between burnout and demographic factors such as age, gender, marital status, and housing.

**Table 3:** Results of multiple logistic regression examining factors affecting rates of burnout, showing increased rates in respiratory therapists and decreased rates in Filipino respondents. * p < 0.05. For categorical values (e.g., age group and gender), the results are presented as log-odds differences compared to the stated reference group. For continuous variables (e.g., COVID-19 work hours per day), the results are presented as a coefficient representing the log-increase in likelihood of burnout per unit increase in the variable.

Factors	Overall burnout (log-odds difference/ratio)	Std. error	p-value
Age			
21-30	Reference		
31-40	-0.570	0.813	0.483
41-50	-1.12	1.49	0.453
51-60	NA	NA	1.00
61-70	2.28	3393	0.9995
Gender			
Female	Reference		
Male	0.0665	0.794	0.933
Ethnicity			
Chinese	Reference		
Malay	-1.50	1.08	0.1643
Indian	0.736	2.30	0.750
Filipino	-1.85	0.900	0.0404*
Marital status			
Single	Reference		
Attached	-1.23	1.17	0.292
Married	-0.912	0.822	0.268
Separated	-3.45	2.68	0.197
Housing			
Public housing	Reference		
Condominium	-1.01	1.18	0.390
Landed housing	-0.0380	1.30	0.977
Occupation			
Nurse	Reference		
Doctor	-2.25	2.15	0.295
Respiratory therapist	4.27	1.89	0.0240*
Patient service associate	-19.9	2399	0.993
Vacation days (per year)	0.0158	0.0374	0.673
COVID-19 work hours (average per day)	-0.163	0.269	0.544
Work scope change (during COVID-19)			
Yes	Reference		
No	-0.0284	0.942	0.976
ICU work			
Yes	Reference		
No	-0.437	0.848	0.607
Shift work			
Yes	Reference		
No	1.19	2.13	0.577

Stress Evaluation (CPSS)

The overall stress rate was 66/76 (86.8%), with a mean score of 22.8 ± 5.9. Physicians reported the highest stress levels at 14/15 (93.3%), followed by respiratory therapists at 10/10 (100%), and nurses at 41/49 (83.7%), though these differences were not statistically significant (p = 0.541). A subgroup analysis indicated a significant association between stress and housing (p = 0.036), while other demographic factors showed no significant correlations.

Pearson’s correlation showed moderate positive correlations between EE (0.380, p < 0.01), DP (0.337, p = 0.033), and CPSS scores, while PA had no significant correlation (0.003, p = 0.979). Cross-tabulation revealed no strong correlation between stress and burnout (p = 0.652).

Anxiety and Depression (PHQ-4)

The PHQ-4 indicated that 63/76 (82.9%) of HCWs were at risk for anxiety and depression, with 9/76 (11.8%) at severe risk, 25/76 (32.9%) at moderate risk, and 29/76 (38.2%) at mild risk. Among respondents, 35/76 (46.1%) were at risk for anxiety and 34/76 (44.7%) for depression. Individual scores showed no significant associations between anxiety and depression risk with demographic factors. Strong correlations were found between PHQ-4 and MBI-HSS scores, particularly for EE (correlation coefficient: 0.41, p < 0.001) and DP (0.30, p = 0.010). The chi-square test indicated a strong correlation between PHQ-4 positivity and professional burnout (phi = 0.38, p < 0.01).

**Figure 1:**
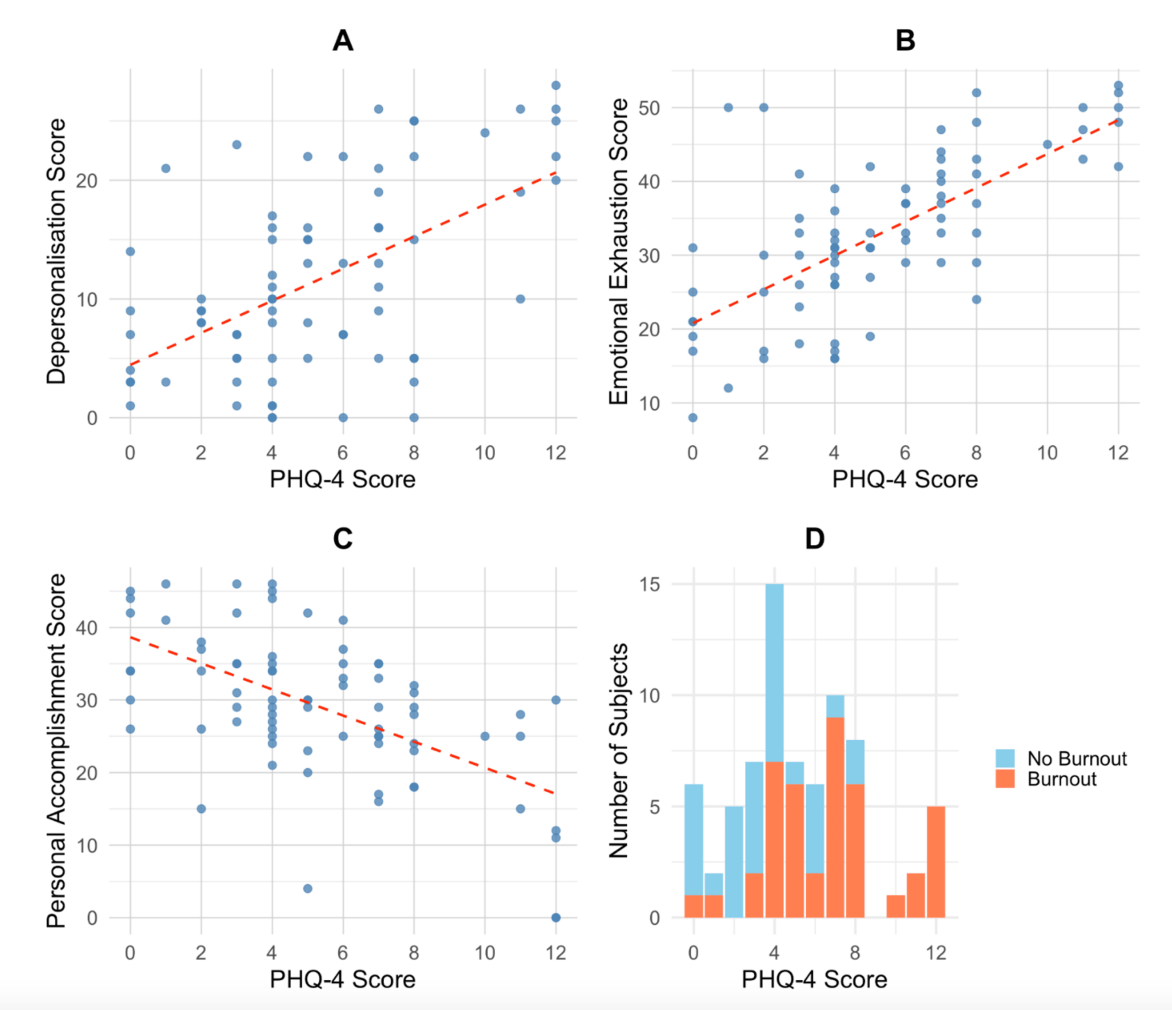
The relationship between Patient Health Questionnaire-4 (PHQ-4) score and DP, EE, and PA scores, as well as overall burnout. A: Relationship between PHQ-4 score and depersonalization (DP) score. B: Relationship between PHQ-4 score and emotional exhaustion (EE) score. C: Relationship between PHQ-4 score and personal accomplishment (PA) score. D: Respondents with and without burnout by PHQ-4 score.

Analysis of individual scores for anxiety and depression revealed no significant associations with demographic factors such as age, gender, religion, marital status, ethnicity, and occupation. Additionally, Pearson’s correlation tests demonstrated strong correlations between burnout, as measured by the MBI-HSS, and anxiety/depression scores from the PHQ-4. The correlation coefficients were 0.30 (p = 0.010) for DP, 0.41 (p < 0.001) for EE, and -0.274 (p = 0.020) for PA. Figure 1 shows the relationship between PHQ-4 score and DP, EE, and PA scores, as well as overall burnout.

## Discussion

This study highlights significant burnout, stress, and mental health challenges among HCWs in Singapore, with notable differences across professional groups. The overall burnout rate of 43/76 (56.6%) underscores the substantial burden faced by the healthcare sector.

Burnout rates among healthcare professionals

Among the groups studied, respiratory therapists (RTs) reported the highest burnout prevalence at 8/10 (80%), followed by nurses and physicians, with this finding remaining significant after multiple logistic regression analysis (p = 0.0404). This aligns with existing literature highlighting RTs as a particularly vulnerable group, with burnout prevalence of up to 75-79% and associations with increased repression and decreased professional fulfillment [22]. Some contributing factors identified included inadequate staffing, excessive workload, and suboptimal leadership [23]. In our institution, unlike nurses and physicians, RTs often work across multiple hospital units, with a higher patient load and constantly changing team dynamics. These factors may contribute to the findings of RTs being disproportionately affected by burnout.

However, it is important to recognize that the smaller sample size of AHP respondents may predispose to an overestimation of burnout prevalence due to statistical variability. In smaller groups, fluctuations or extreme cases can disproportionately affect the results, potentially introducing bias into the findings.

A detailed examination of burnout components (DP, EE, and PA) offers additional insights into how burnout manifests across different healthcare professions. The prevalence of DP at 35/76 (46.1%), with a mean score of 11.6, suggests a considerable degree of detachment in patient and colleague interactions across HCWs. The high prevalence of PA burnout at 64/76 (84.2%) reflects a common perception of reduced professional achievement, which could be especially relevant in settings with high patient turnover and complex care needs, potentially impacting motivation and job satisfaction. EE, a core element of burnout, was prevalent among 53/76 (69.7%) of respondents, with nurses reporting the highest EE scores. This may reflect the emotional toll and demanding nature of patient care responsibilities in nursing. Similarly, Moll et al. found a significant increase in burnout among ICU nurses, with rates rising from 58% pre-pandemic to 72% during the pandemic, and EE showing marked increases as well [24].

Burnout rates across other demographic variables

In examining demographic and occupational factors associated with burnout, multiple logistic regression revealed that Filipino ethnicity was significantly associated with reduced levels of burnout (p = 0.0404). This is in contrast with broader evidence showing high burnout rates among Filipino HCWs. A multi-country study found that 20% experienced burnout, linked to long hours, high job risk, and inadequate protective equipment, including HCWs in the Philippines [25]. Another study on primary care improvements in the Philippines showed that while some interventions boosted job satisfaction, burnout remained prevalent, especially in remote areas [26]. These differences may be due to variations in study populations, methodological approaches, and potential protective factors. Our analysis adjusted for key demographic and occupational variables. Furthermore, cultural resilience, workplace support, or selection effects may explain our findings. Rather than contradicting broader evidence, our findings highlight the complexity of burnout and suggest that Filipino ethnicity, within our study context, was associated with lower burnout levels after adjusting for key demographic and occupational variables.

Stress, anxiety, and depression

The overall stress rate of 66/76 (86.8%) in the study sample, along with particularly high rates among physicians and AHPs, reflects a substantial burden of occupational stress. Although differences among professional groups were not statistically significant, the subgroup analysis revealed a significant association between stress and housing (p = 0.036), which may point to the influence of environmental and lifestyle factors on stress levels.

The moderate positive correlations observed between stress (as measured by CPSS scores) and key burnout dimensions (EE and DP) underscore the close link between stress and burnout among HCWs. Specifically, a correlation of 0.380 between CPSS scores and EE (p < 0.010) and 0.337 with DP (p = 0.033) indicates that increased perceived stress is associated with greater levels of EE and DP, two central components of burnout. These findings suggest that stress serves as a substantial driver of burnout, supporting the view that reducing workplace stressors could play a critical role in mitigating burnout among HCWs.

The significant association between stress and burnout is consistent with existing literature. For example, Carpi et al. identified perceived stress as a major predictor of both EE and DP among healthcare professionals, suggesting that stress contributes directly to HCWs’ mental and emotional depletion, potentially impairing their ability to engage with patients and perform effectively [27]. Additionally, Makara-Studzińska et al. documented similar findings in a study of emergency dispatchers, where stress was shown to predict EE and DP, especially amid the acute demands of the COVID-19 pandemic [28]. This relationship between stress and burnout was potentially amplified during the pandemic due to increased workloads, high patient acuity, and the emotional toll of managing critical cases.

Lastly, mental health outcomes, as measured by the PHQ-4, further underscore the psychological challenges faced by HCWs, with 63/76 (82.9%) participants at risk for anxiety and depression, and 9/76 (11.8%) at severe risk. Notably, the strong correlations between PHQ-4 scores and MBI-HSS burnout scores, particularly for EE (0.41, p < 0.001) and DP (0.30, p = 0.010), highlight a close relationship between burnout and mental health. The significant association between PHQ-4 positivity and burnout (phi = 0.38, p < 0.010) further reinforces this link. This is consistent with findings from multiple studies. For instance, Pappa et al. reported significant levels of depression and anxiety among HCWs, with 52% experiencing moderate/severe EE and 19.5% experiencing moderate/severe DP [29]. Similarly, Pala et al. found that nurses and other non-physician staff had higher odds of screening positive for depression and anxiety, which were strongly associated with burnout [30]. No significant associations were found between mental health risks and demographic variables, suggesting that mental health challenges are pervasive and not necessarily influenced by factors such as age, gender, or marital status.

Strengths and limitations

Our study provides detailed insights into the different manifestations of burnout and psychological distress by examining all three primary burnout components (DP, EE, and PA) across healthcare professions. While largely consistent with existing literature, differences such as lower rates of burnout among Filipino HCWs as well as higher burnout rates among respiratory therapists highlight the importance of taking an individualized approach to burnout management in healthcare settings.

At the same time, there are limitations that should be acknowledged. First, the survey was conducted in a single center, which may affect the generalizability of the findings to other healthcare settings or regions. The unique characteristics of the Singaporean healthcare system and cultural context may influence burnout and stress levels differently than in other countries. A small sample size also limits the statistical power of our findings and generalizability. A larger, more diverse sample could provide more nuanced insights, especially in terms of the differences in challenges faced between healthcare worker groups. For instance, AHPs other than respiratory therapists (such as physiotherapists, occupational therapists, and speech therapists) were not represented in our sample due to a lack of responses.

Secondly, significant time has elapsed since the COVID-19 pandemic and our survey prior to publication. While similar studies have been done in the meantime, limiting the novelty of our findings, there is a greater breadth of literature that informs our discussion and provides important context for us to discuss factors that may be more unique to our local context.

Thirdly, our research design was cross-sectional, which limits the ability to establish causal relationships between burnout, stress, and mental health outcomes. Longitudinal studies can provide more insight into how these factors interact over time and their long-term effects on HCWs.

Fourthly, our study relied on self-reported measures for assessing burnout, stress, and mental health conditions. This may potentially introduce response bias, as participants may underreport or overreport their experiences based on various factors, including social desirability or fear of stigma. This was mitigated by allowing respondents to submit questionnaires completely anonymously, as described in our methods.

Lastly, aside from demographic factors, there may be other confounding factors that were not accounted for. Some hypothesized factors include workplace culture, specific job roles, or individual coping mechanisms. Future studies should explore conceptual frameworks that could help to incorporate these factors into a model to provide a more comprehensive understanding of the predictors of burnout among HCWs.

Future direction

Future research should focus on longitudinal studies to track burnout, stress, and mental health over time, particularly in the post-pandemic period, to better understand burnout’s persistence and evolution. Examining the effectiveness of protective factors for respiratory therapists, such as adequate staffing and community support, could inform targeted interventions for other high-risk groups. Implementing stress reduction strategies and mental health support across healthcare settings, particularly in high-stress environments like ICUs and emergency departments, may help alleviate burnout and enhance the well-being of healthcare workers.

## Conclusions

In conclusion, despite the limitations discussed, this study highlights the urgent need for targeted interventions to address burnout and mental health issues among HCWs in Singapore. It is imperative that healthcare organizations implement comprehensive support strategies, including mental health resources, stress management programs, and community-building initiatives, to bolster the resilience of their staff.

Recognizing and addressing the psychological toll of the pandemic is essential not only for the well-being of HCWs but also for the overall quality of patient care in this critical time. Future research should focus on longitudinal studies to explore the long-term effects of the pandemic on healthcare professionals' mental health and the effectiveness of implemented support systems.

## References

[1] (2025). World Health Organization. International Statistical Classification of Diseases and Related Health Problems (ICD). https://www.who.int/standards/classifications/classification-of-diseases.

[2] Maslach C, Leiter MP (2016). Understanding the burnout experience: recent research and its implications for psychiatry. World Psychiatry.

[3] Hodkinson A, Zhou A, Johnson J (2022). Associations of physician burnout with career engagement and quality of patient care: systematic review and meta-analysis. BMJ.

[4] Li LZ, Yang P, Singer SJ, Pfeffer J, Mathur MB, Shanafelt T (2024). Nurse burnout and patient safety, satisfaction, and quality of care: a systematic review and meta-analysis. JAMA Netw Open.

[5] Ryan E, Hore K, Power J, Jackson T (2023). The relationship between physician burnout and depression, anxiety, suicidality and substance abuse: a mixed methods systematic review. Front Public Health.

[6] John A, Bouillon-Minois JB, Bagheri R (2024). The influence of burnout on cardiovascular disease: a systematic review and meta-analysis. Front Psychiatry.

[7] Denning M, Goh ET, Tan B (2021). Determinants of burnout and other aspects of psychological well-being in healthcare workers during the COVID-19 pandemic: a multinational cross-sectional study. PLoS One.

[8] Ghio L, Patti S, Piccinini G, Modafferi C, Lusetti E, Mazzella M, Del Sette M (2021). Anxiety, depression and risk of post-traumatic stress disorder in health workers: the relationship with burnout during COVID-19 pandemic in Italy. Int J Environ Res Public Health.

[9] Zheng Y, Tang PK, Lin G, Liu J, Hu H, Wu AM, Ung CO (2023). Burnout among healthcare providers: its prevalence and association with anxiety and depression during the COVID-19 pandemic in Macao, China. PLoS One.

[10] Chandawarkar A, Chaparro JD (2021). Burnout in clinicians. Curr Probl Pediatr Adolesc Health Care.

[11] Papazian L, Hraiech S, Loundou A, Herridge MS, Boyer L (2023). High-level burnout in physicians and nurses working in adult ICUs: a systematic review and meta-analysis. Intensive Care Med.

[12] Coimbra BM, Zylberstajn C, van Zuiden M, Hoeboer CM, Mello AF, Mello MF, Olff M (2023). Moral injury and mental health among health-care workers during the COVID-19 pandemic: meta-analysis. Eur J Psychotraumatol.

[13] Al Maqbali M, Alsayed A, Hughes C, Hacker E, Dickens GL (2024). Stress, anxiety, depression and sleep disturbance among healthcare professional during the COVID-19 pandemic: an umbrella review of 72 meta-analyses. PLoS One.

[14] Guttormson JL, Calkins K, McAndrew N, Fitzgerald J, Losurdo H, Loonsfoot D (2022). Critical care nurse burnout, moral distress, and mental health during the COVID-19 pandemic: a United States survey. Heart Lung.

[15] Estephan L, Pu C, Bermudez S, Waits A (2023). Burnout, mental health, physical symptoms, and coping behaviors in healthcare workers in Belize amidst COVID-19 pandemic: a nationwide cross-sectional study. Int J Soc Psychiatry.

[16] Teo I, Chay J, Cheung YB (2021). Healthcare worker stress, anxiety and burnout during the COVID-19 pandemic in Singapore: a 6-month multi-centre prospective study. PLoS One.

[17] Tan BY, Kanneganti A, Lim LJ (2020). Burnout and associated factors among health care workers in Singapore during the COVID-19 pandemic. J Am Med Dir Assoc.

[18] von Elm E, Altman DG, Egger M, Pocock SJ, Gøtzsche PC, Vandenbroucke JP (2007). The Strengthening the Reporting of Observational Studies in Epidemiology (STROBE) statement: guidelines for reporting observational studies. PLoS Med.

[19] Shi Y, Gugiu PC, Crowe RP, Way DP (2019). A Rasch analysis validation of the Maslach Burnout Inventory-Student Survey with preclinical medical students. Teach Learn Med.

[20] Cohen S, Kamarck T, Mermelstein R (1983). A global measure of perceived stress. J Health Soc Behav.

[21] Kroenke K, Spitzer RL, Williams JB, Löwe B (2009). An ultra-brief screening scale for anxiety and depression: the PHQ-4. Psychosomatics.

[22] Roberts KJ, Silvestri JA, Klaiman T (2022). Well-being among respiratory therapists in an academic medical center during the COVID-19 pandemic. Respir Care.

[23] Miller AG, Roberts KJ, Smith BJ (2021). Prevalence of burnout among respiratory therapists amid the COVID-19 pandemic. Respir Care.

[24] Moll V, Meissen H, Pappas S (2022). The coronavirus disease 2019 pandemic impacts burnout syndrome differently among multiprofessional critical care clinicians—a longitudinal survey study. Crit Care Med.

[25] Teo I, Nadarajan GD, Ng S (2022). The psychological well-being of Southeast Asian frontline healthcare workers during COVID-19: a multi-country study. Int J Environ Res Public Health.

[26] De Mesa RY, Marfori JR, Fabian NM (2023). Experiences from the Philippine grassroots: impact of strengthening primary care systems on health worker satisfaction and intention to stay. BMC Health Serv Res.

[27] Carpi M, Bruschini M, Di Vito A, Burla F (2024). Burnout and perceived stress among Italian physical therapists during the COVID-19 pandemic: a cross-sectional study. Psychol Health Med.

[28] Makara-Studzińska M, Załuski M, Adamczyk K (2021). Polish emergency dispatchers during a COVID-19 pandemic - burnout syndrome, perceived stress, and self-efficacy. Effects of multidimensional path analysis. Front Psychol.

[29] Pappa S, Barnett J, Berges I, Sakkas N (2021). Tired, worried and burned out, but still resilient: a cross-sectional study of mental health workers in the UK during the COVID-19 pandemic. Int J Environ Res Public Health.

[30] Pala AN, Chuang JC, Chien A (2022). Depression, anxiety, and burnout among hospital workers during the COVID-19 pandemic: a cross-sectional study. PLoS One.

